# Special Issue: Resistance to Targeted Therapies in Human Cancer

**DOI:** 10.3390/biomedicines11020414

**Published:** 2023-01-31

**Authors:** Tae-Won Lee, Hee-Joo Choi, Kyung-Min Lee, Jeong-Yeon Lee

**Affiliations:** 1 Department of Life Science, College of Natural Sciences, Hanyang University, Seoul 04763, Republic of Korea; 2 Department of Pathology, College of Medicine, Hanyang University, Seoul 04763, Republic of Korea; 3 Hanyang Biomedical Research Institute, Hanyang University, Seoul 04763, Republic of Korea; 4 Hanyang Institute of Bioscience and Biotechnology, Hanyang University, Seoul 04763, Republic of Korea; 5 Research Institute for Natural Sciences, Hanyang University, Seoul 04763, Republic of Korea; 6 Research Institute for Convergence of Basic Sciences, Hanyang University, Seoul 04763, Republic of Korea

Cancer is the second leading cause of death worldwide, accounting for approximately 10 million deaths in 2020. A huge number of effective target therapies have been developed over the past few decades for cancer treatment, which in turn have had the effect of improving the patients’ prognoses. However, most cancer patients eventually acquire resistance to these therapies, which is one of the main causes of death in cancer patients. This Special Issue, entitled “Resistance to Targeted Therapies in Human Cancer”, which includes five original research articles and four review articles, shares insights on the mechanisms of targeted therapy resistance, discusses current limitations, and finally suggests novel strategies to overcome.

Small molecule kinase inhibitors targeting key oncogenic signaling pathways are the most widely used anti-cancer drugs in various types of human cancer. Unfortunately, innate and acquired resistance to these targeted therapies is very common and difficult to overcome due to the diverse and complex mechanisms of resistance. Taylor and colleagues [1] comprehensively reviewed the different types of small-molecule inhibitors that are currently available as cancer-targeted drugs, such as selective kinase inhibitors and nonselective multi-kinase inhibitors, and their known resistance mechanisms. They also discussed the advantages and disadvantages of these single- and multi-kinase inhibitors for cancer treatment. Among the multi-kinase inhibitors, sunitinib, which targets vascular endothelial growth factor (VEGF) and other tyrosine kinases, is currently used as a first-line therapy for the treatment of renal cell carcinoma (RCC). However, the patients often develop resistance to sunitinib, whereas a second-line therapy for the treatment-resistant RCC has not yet been established. A research article by Juengel et al. [2] compared the antiangiogenic properties of other multi-kinase inhibitors targeting VEGF, such as axitinib and sorafenib, after the generation of sunitinib-resistant human endothelial cells (HUVECs). Given the remarkable inhibitory effect of axitinib and sorafenib on angiogenesis in the sunitinib-resistant HUVECs, it can be speculated that these multi-kinase inhibitors may be useful as second-line treatment options in RCC resistant to sunitinib.

The tumor microenvironment (TME) refers to the environment around a tumor, including the extracellular matrix (ECM), blood vessels, immune cells, adipocytes, and cancer-associated fibroblasts (CAFs) [3]. In recent years, several studies have revealed that the TME is one of the critical factors determining the response to various anti-cancer therapies. Mollah and Varamini [4] reviewed breast cancer-associated fibroblasts (BCAFs) that play a crucial role in resistance to therapy in triple-negative breast cancers (TNBCs) in this issue. The authors highlighted that BCAFs that are mainly derived from mesenchymal stem cells and adipocytes share genetic and phenotypic characteristics similar to CAFs. In addition, they summarized therapeutic strategies targeting BCAFs, including small molecules, nucleic acid-based agents, antibodies, peptides, and nanoparticles. Finally, they addressed its current limitation, that is, the lack of a standardized method to capture BCAF, and thus reminded us of the need to discover potential biomarkers to evaluate BCAF. On the other hand, the complex and diverse immune context present in the tumor microenvironment can also influence on response to therapy [5]. Arendt et al. [6] proposed that immune cells, especially myeloid cells, are required for the adequate evaluation of Kirsten rat sarcoma virus (KRAS) blockade efficacy. Global transcriptome analysis identified *KRAS*^MUT^-driven overexpression of interleukin-1 receptor 1 (Il1r1) and C-C-motif chemokine ligand 2 (Ccl2), potentially participating in a signaling loop sustaining tumorigenicity. Furthermore, they verified that the paracrine loop between *KRAS*^MUT^ cancer cells and myeloid cells is required for a significant response to KRAS blockade in vivo. This study addresses the limitations of conventional in vitro drug screening and thus provides a rationale for a screening approach that considers the tumor immune microenvironment.

Including the myeloid cells mentioned above, various types of immune cells exist in the TME, and their diversity and immunogenicity are closely associated with the response to immune checkpoint blockade (ICB) in cancer patients. Kim et al. [7] summarized diverse predictive biomarkers for ICBs in TNBC, such as tumor mutational burden, neoantigens, PD-L1 expression, and tumor-infiltrating lymphocytes (TILs). Moreover, they divided the mechanisms of resistance acquisition by cancer-cell-intrinsic and -extrinsic factors. In addition to well-known oncogenic factors such as *MYC* and the MAPK pathway, novel checkpoints, cytokines, and metabolites were also referred to as putative drivers of the mechanism of resistance to ICB. Finally, they discussed the validity of approaches that can construct a hot TME for overcoming resistance to ICB. As mentioned in Kim and colleagues’ review, TILs and antitumor immunity of TME are key determinants for predicting the response to ICB. Given the need for a hot TME construct to overcome resistance to ICB, the activation of specific signaling pathways that can enhance the innate immune response is a remarkable strategy to improve response rates to ICB. In line with this idea, the stimulator of interferon genes (STING) that stimulate the innate immune response is a fascinating target. The STING pathway has been shown to boost innate immune response through triggering tank-binding kinase 1 (TBK1)–interferon regulatory factor 3 (IRF3) signaling axis [8]. A high-throughput screening performed by Jung and colleagues [9] identified KAS-08, a novel small molecule augmenting the expression of interferon-stimulated genes selectively in presence of cyclic GMP-AMP (cGAMP). They found that the KAS-08 in combination with cGAMP significantly increases the release of inflammatory cytokines, Interferon-β and IP-10. Finally, the combination treatment of KAS-08 with cGAMP elicited a significant anti-tumor effect compared to single cGMP treatment in vivo. Although the efficacy of the combined treatment of KAS-08 with ICB has not been verified through this study, it is expected that the combination will show a significant synergistic effect.

Because of the plasticity of epigenetic modifications, epigenetic regulators that are frequently dysregulated in human cancers have been considered as attractive cancer targets to overcome resistance to conventional cancer therapies [10]. Recently, the bromodomain and extraterminal domain (BET) family of proteins, which are crucial epigenetic readers, have emerged as potential therapeutic targets for human leukemia and solid tumors. Braun and colleagues [11] reported the biological consequences of the anti-tumor effect of BET inhibitors (BETi) in NPM1-mutated (NPM1c) acute myeloid leukemia (AML) cells. They found that BETi, OTX015 (MK-8628), and JQ1 were able to induce differentiation and apoptosis via the proteasomal degradation of cytosolic NPM1c in NPM1c AML cells and patient-derived bone marrow blasts, whose anti-tumor effects were comparable to those of all trans-retinoic acid (ATRA) plus arsenic trioxide (ATO) combination therapy. These results provide a rationale for the need for clinical trials of BETi in patients with NPM1c AML who develop therapeutic resistance.

Targeting protein degradation could be an alternative therapeutic strategy for currently unavailable cancer targets. Zou et al. [12] demonstrated that the proteasomal degradation of ABCB1, a multidrug resistance (MDR) protein, by the natural compound rutaecarpine can improve sensitivity to chemotherapeutic agents in drug-resistant tumors. In ABCB1-overexpressing breast and lung cancer cells, rutaecarpine treatment reduced the protein levels of ABCB1 via the MARCH8-mediated ubiquitin-proteasome pathway, thereby facilitating intracellular drug accumulation and adriamycine/paclitaxel-induced apoptosis. Recent advances in targeted protein degradation (TPD) technology are expected to provide a great opportunity to effectively control cancer targets, for which it has been difficult to develop inhibitors in the past. Kim and colleagues [13] reviewed the general function, advantages, and current challenges of proteolysis targeting chimera (PROTAC), one of the major TPD molecules that can hijack the E3 ubiquitin ligase and recruit a target protein for degradation, in the treatment of cancer. Considering the advantage of PROTAC technology that is able to target both enzymatic and non-enzymatic proteins, this approach could be a potent and promising alternative strategy for traditional undruggable cancer targets.

In summary, the articles published in this Special Issue outline the mechanisms by which resistance is acquired at the molecular-to-cellular level, as well as the mechanisms of resistance to small-molecule and large-molecular protein drugs. The research articles propose a novel compound or an approach as a countermeasure based on the molecular mechanisms of resistance. In the review articles, current limitations and promising strategies are discussed in depth. These series of articles are anticipated to contribute to providing a rationale for the initiation of related clinical studies.
